# Decoration Day

**Published:** 1889-06

**Authors:** 


### HALL'S JOURNAL OF HEALTH

TRUTH DEMANDS NO SACRIFICE: ERROR CAN MAKE NONE.

VOL. 36. JUNE, 1889. No. 6.

### SPECIAL.

We shall send, during the present month, to each subscriber in arrears a bill of his indebtedness, and earnestly request that the amount be remitted to as without further delay. It is needed in carrying forward our work.

### DECORATION DAY.

The thirtieth day of May has been set apart as one consecrated to the patriotic dead, not alone the leaders and head men in a great conflict of arms, but to all alike who served and fell in a struggle to preserve the national unity and the national honor, wherein the real heroes were the men who volunteered to do a soldier's duty in the ranks, and offered up their lives that their country might live. There was no vain glory in this act, no expectation of reward beyond the appreciation of a generous people of the self-sacrificing patriotism which prompted it.

It is the survivors of the conflict who meet on this memorial day to make their heart offerings and revive the memories of old time comradship on many a weary march and well fought field. The day has been well chosen, for it is the season of flowers in all their freshness and beauty, and flowers have been chosen as the fittest offering to be laid upon the humble hillocks that mark the burial places of the heroic dead whose remains have been gathered to consecrated ground. But the day and the tribute should be for all; all who fell in the conflict, for the hands that clutched the sword in intersectional strife are clasped in friendship now, let us hope, for all time. The victory too, was for all, for even the States in revolt have reason to rejoice with their northern sisters that the cause which gave rise to the hot blood of contention, has been swept forever away. Defeat was for them a victory in all that elevates a people and makes a nation great, a truth which they realize now as never before.

Why should not the day be common to both north and south; the flowers laid by loving hands upon heroic graves, a peace offering and a bond for all time to come?

So may it be.

